# The effect of modifiable risk factors on geographic mortality differentials: a modelling study

**DOI:** 10.1186/1471-2458-12-79

**Published:** 2012-01-25

**Authors:** Christopher E Stevenson, Haider Mannan, Anna Peeters, Helen Walls, Dianna J Magliano, Jonathan E Shaw, John J McNeil

**Affiliations:** 1 School of Public Health and Preventive Medicine, Monash University, The Alfred Centre, 99 Commercial Road, Melbourne, Victoria, Australia; 2 Baker IDI Heart and Diabetes Institute, The Alfred Centre, 99 Commercial Road, Melbourne, Victoria, Australia

## Abstract

**Background:**

Australian mortality rates are higher in regional and remote areas than in major cities. The degree to which this is driven by variation in modifiable risk factors is unknown.

**Methods:**

We applied a risk prediction equation incorporating smoking, cholesterol and blood pressure to a national, population based survey to project all-causes mortality risk by geographic region. We then modelled life expectancies at different levels of mortality risk by geographic region using a risk percentiles model. Finally we set high values of each risk factor to a target level and modelled the subsequent shift in the population to lower levels of mortality risk and longer life expectancy.

**Results:**

Survival is poorer in both Inner Regional and Outer Regional/Remote areas compared to Major Cities for men and women at both high and low levels of predicted mortality risk. For men smoking, high cholesterol and high systolic blood pressure were each associated with the mortality difference between Major Cities and Outer Regional/Remote areas--accounting for 21.4%, 20.3% and 7.7% of the difference respectively. For women smoking and high cholesterol accounted for 29.4% and 24.0% of the difference respectively but high blood pressure did not contribute to the observed mortality differences. The three risk factors taken together accounted for 45.4% (men) and 35.6% (women) of the mortality difference. The contribution of risk factors to the corresponding differences for inner regional areas was smaller, with only high cholesterol and smoking contributing to the difference in men-- accounting for 8.8% and 6.3% respectively-- and only smoking contributing to the difference in women--accounting for 12.3%.

**Conclusions:**

These results suggest that health intervention programs aimed at smoking, blood pressure and total cholesterol could have a substantial impact on mortality inequities for Outer Regional/Remote areas.

## Background

Mortality rates in Australia are higher in rural and remote areas than major cities. In 2002-04, death rates for males in Inner Regional, Outer Regional, Remote and Very Remote areas were significantly higher than their counterparts in Major Cities, with standardised mortality ratios (SMR) of 1.1, 1.2, 1.2 and 1.7 respectively. Females in these areas also had significantly higher death rates, with SMRs of 1.1, 1.1, 1.2 and 1.7 respectively [1]. This could be due to a range of influences including lower socioeconomic status, poorer risk factor profiles, differential access to health services and the high proportion of Indigenous people in these areas [1-3]. Research in other countries has shown that the drivers of geographic variation in mortality include societal and economic factors but that individual health risk factors play a significant role [4-7].

The Australian Human Rights Commission identified rural health inequities as a substantial human rights issue for Australia [8]. Reduction of risk factor prevalence across regions may be one way to address these inequities, but the potential of this approach is unknown. Here we examine the extent to which variation in life expectancy between Australian geographic regions is associated with variations in selected modifiable risk factors. We adapt a previously developed coronary heart disease model developed for the Australian population to analyse the potential impact of changing the prevalence of smoking, high cholesterol and high blood pressure on life expectancy in the Major City, Inner Regional and combined Outer Regional/Remote regions in Australia [9].

## Methods

We used a mortality risk prediction equation applied to a national population survey to model percentiles of short-term mortality risk by geographic region. We then modelled survival curves and associated life expectancies for each percentile of risk. Finally we modelled the effect of each risk factor by setting high values of that factor to a 'target' level and modelling the subsequent population shift to lower percentiles of mortality risk and associated longer life expectancy.

### Geographic regions

This study used the Australian Standard Geographical Classification (ASGC), which groups geographic areas into five regions--defined using an index of the remoteness of a location from the services provided by large towns or cities. The regions are Major City, Inner Regional, Outer Regional, Remote and Very Remote [10]. The Major City region contains urban centres with a population greater than 250,000. Conversely the Very Remote region is defined to contain the most remote one per cent of the population while the Remote and Very Remote regions together contain the most remote three per cent of the population. According to the 2001 Australian population census, the Inner Regional regions together contained 3,872,693 people, the Outer Regional regions together contained 1,978,495 people, the Remote regions together contained 334,683 people and the Very Remote regions together contained 201,120 people [1].

Our study used three regions--Major City, Inner Regional and a combined Outer Regional/Remote group.

### Data sources

#### The AusDiab study

Our national population survey was the baseline survey of the Australian Diabetes, Obesity and Lifestyle (AusDiab) study [11]. This was a cross-sectional, national, population-based survey of 11,247 adults aged ≥ 25 years in 1999-2000, comprising a household interview and biomedical examination at a testing site. Among the information collected was seated systolic and diastolic blood pressure; self-reported cigarette smoking status; and serum total cholesterol. Of the 11,247 survey participants 8,706 were aged 40 or over. Of these 8,534 had complete data for systolic and diastolic blood pressure; self-reported cigarette smoking status; and serum total cholesterol--3,880 male and 4,654 female.

Because of small numbers we grouped the oldest AusDiab participants into one group aged 75 years and over. The percentile scores for this group were taken to apply equally to the age groups 75-79, 80-84 and 85 years and over.

The number of AusDiab participants in Major Cities, Inner and Outer Regional and Remote areas aged 40 and over and the number with complete risk factor data for systolic and diastolic blood pressure; self-reported cigarette smoking status; and serum total cholesterol are given in Table 1. There were no AusDiab collection centres in Very Remote areas and only one collection centre in a Remote area, so we excluded Very Remote areas and combined the Outer Regional and Remote areas.

**Table 1:** AusDiab sample counts and number with complete data for blood pressure; self-reported cigarette smoking status; and serum total cholesterol in each region for respondents aged 40 and over by age and sex

Region								
**Age**	**Major city**		**Inner regional**		**Outer regional**		**Total**	

	***Sample count***	***Complete data***	***Sample count***	***Complete data***	***Sample count***	***Complete data***	***Sample count***	***Complete data***

		*Males*						
40-45	242	240	147	144	208	203	597	587
45-49	270	267	176	176	217	209	663	652
50-54	314	310	171	170	197	194	682	674
55-59	248	245	121	115	129	126	498	486
60-64	210	206	107	104	112	111	429	421
65-69	206	201	91	88	101	100	398	389
70-74	138	133	106	100	89	85	333	318
75 and over	186	181	86	84	90	88	362	353

Total	1,814	1,783	1,005	981	1,143	1,116	3,962	3,880

		*Females*						
40-45	357	349	212	206	244	244	813	799
45-49	351	341	223	220	229	227	803	788
50-54	346	339	196	193	201	196	743	728
55-59	296	291	142	138	171	170	609	599
60-64	263	260	105	104	120	118	488	482
65-69	185	184	125	120	126	125	436	429
70-74	192	189	110	105	99	97	401	391
75 and over	219	215	115	108	117	115	451	438

Total	2,209	2,168	1,228	1,194	1,307	1,292	4,744	4,654

#### National population and death counts

National population and death counts classified by age, sex and geographic region for 2001 - 2006 were supplied by the Australian Institute of Health and Welfare (AIHW). The population counts were derived from the Australian Bureau of Statistics (ABS) mid-year population estimates. Deaths data were derived from the AIHW National Mortality Database comprising all deaths registered in Australia.

#### Availability of data

The AusDiab confidentialised unit record data are available for serious scientific research proposals that are consistent with the overall AusDiab program of research activities; that do not conflict with work in progress; and where the interests and personal privacy of survey subjects are protected and Institutional Ethics Committee approval has been given for the proposed research. Access to the data is decided by the AusDiab Scientific Research Committee on the basis of a written application.

National population and death counts classified by age and sex are freely available from the AIHW web-site. However, data with more detailed classifications, such as the regional classifications used here, are available on written application from a researcher provided they are aggregated to levels where there is no potential for identification of data for individuals. Where such specific tabulations are requested, the AIHW charges a fee based on full-cost recovery for any programming and other costs associated with the planning, extraction and provision of the data.

### Risk prediction equation

The basis of the categorisation of mortality risk into percentiles was a risk prediction equation for cardiovascular disease mortality using modifiable health risk factors. We used an index developed by the SCORE (Systematic COronary Risk Evaluation) project [12]. This project assembled a pooled dataset of cohort studies from 12 European countries and derived a risk prediction equation for risk of death from cardiovascular disease. The pooled data set comprised 88,080 women and 117,098 men and the risk was predicted for ages 45 - 64. The equation was calculated separately for men and women and incorporated age as the basis of the hazard function. The variables in this equation are: smoking status, total cholesterol and systolic blood pressure (SBP).

To assess the suitability of the SCORE statistic as a proxy for short-term all-cause mortality risk for our study we followed a similar method to Aktas et al [13]. The baseline AusDiab sample has been followed up for subsequent death via a regular linking with Australian death registry data, commencing with a follow-up to 2004 and then followed up annually with the most recent linkage being for 2008 [14]. We predicted the risk of death using a Cox proportional hazards regression equation with the SCORE statistic as a predictor variable and death from any cause up to 1 June 2004 as the outcome variable. Follow-up to 2004 was chosen rather than follow-up to 2008 because the model uses the SCORE statistic to allocate short-term mortality. The model was fitted separately for men and women and included age categorised into five year age groups as a covariate. SCORE was originally derived to predict risk at ages 45 - 64, but our study applies to ages 40 and over. So we fitted the Cox regression to ages 40 and over to demonstrate SCORE's overall ability to predict all cause mortality risk and we fitted a separate regression to ages 65 and over to demonstrate its predictive ability at older ages. We used the adjusted Hosmer-Lemeshow goodness of fit statistic and the Harrell's C statistic to assess the regression equations' fit and predictive ability [15,16].

### The risk percentiles model

A summary of the modelling methods is presented here. A detailed description of these methods is included in the additional file 1: appendix.

The risk percentiles model was used to derive an average life expectancy at each percentile of projected mortality risk based on the SCORE equation. The Australian disease burden associated with the modifiable risk factors incorporated in our risk prediction equation is very small below age 40, so our modelling was applied to ages 40 and over [17]. The steps involved in the calculation, within each age, sex and regional group, are as follows [9]:

1. Divide the Australian population into mortality risk percentiles.

The SCORE equation was applied to the AusDiab data to calculate the five-year probability of death for each individual in the survey (their *risk score*). Survey weights were applied to the survey sample so that the data could be taken as estimates for the total Australian population and the values of the risk score that divide the weighted sample into percentiles were then calculated. Each survey participant was allocated to a risk percentile using their risk score and a total risk score for all participants calculated for each percentile.

2. Allocate deaths to risk percentiles.

The national count of deaths summed across 2001 - 2006 was allocated to the risk percentiles using the ratios of the aggregate risk score between percentiles. For example, if one percentile group had a total risk score twice that of another group then the deaths were allocated between them in the ratio 2 - 1. When divided by the population count summed across 2001 - 2006 (assumed evenly spread between risk percentiles) this gives us the death rate for each risk percentile group.

3. Use these mortality rates to construct sex-specific life tables and survival curves for each risk percentile within each regional group.

After step 2, we had a set of age specific mortality rates by sex for each percentile group within each region. We applied standard life table techniques [18] using these mortality rates to construct a sex-specific life table for each percentile group within each region. We used these life tables to generate sex-specific survival curves and life expectancies for each percentile group within each region.

### Modelling the effect of risk factors on inter-regional mortality differentials

The effect of risk factors on inter-regional mortality differentials was modelled for the 2006 Australian population. For each age-sex-region group, the modelled life expectancy at each percentile of projected mortality risk as derived above was multiplied by the corresponding 2006 population count to obtain the projected total life years for each group. This was summed over the percentiles and ages within each region and divided by the regional population count to calculate a *baseline average potential years of life (APYL) per person *for each region.

The risk factors modelled in this paper were those in the SCORE equation which are potentially modifiable--smoking, total cholesterol and systolic blood pressure [12]. We returned to the AusDiab survey data to model the effect of setting high values of each risk factor separately to a 'target' value. For example, in examining blood pressure each survey participant with a SBP greater than or equal to 140 mmHg had his/her risk score recalculated assuming a SBP of 120--creating a synthetic sample of people with SBP below high risk levels. The participants were reallocated to the risk percentiles using the existing risk score cut offs with their revised risk score values. This, when weighted using the AusDiab survey weights, provided a modelled estimate of the shift in the total Australian population between each percentile group under this scenario. Note that this does not imply that any practical intervention would be capable of reducing blood pressure to a target level for all people with high blood pressure. The aim of the model is to investigate a theoretical impact of blood pressure reduction by comparing the mortality outcomes associated with the current population with those projected for a population where no one has a blood pressure above the target value.

We multiplied the existing average life expectancy for each percentile-age-sex-region group by the new population count projected to be in that group under each scenario. This was summed over the percentiles and ages within each region and divided by the regional population count to calculate a *scenario average potential years of life per person *for each region.

This procedure is illustrated in Table 2 with a hypothetical population of 100,000 people divided into risk quartiles (not percentiles) for ease of presentation. Column 2 presents the number of people in each quartile at baseline and column 3 is the average life expectancy from our model for each quartile. Column 4 presents the baseline lifeyears estimate, which is calculated by multiplying column 2 by column 3. If we sum column 4 and divide this by the total population, we get the baseline APYL per person (32.5 years). Column 5 presents the number of people allocated to each quartile after the survey participants have had their risk adjusted as described above. Column 6 represents the scenario lifeyears estimate and is calculated by multiplying column 3 by column 5. If we sum column 6 and divide this by the total population, we get the scenario APYL per person (35.0 years).

**Table 2:** Modelling the impact of a risk factor illustrated with a hypothetical population

Quartile of risk	Baseline population distribution	Average life expectancy	Baseline PYL	Scenario population distribution	Scenario PYL
1	25,000	40	1,000,000	40,000	1,600,000
2	25,000	35	875,000	30,000	1,050,000
3	25,000	30	750,000	20,000	600,000
4	25,000	25	625,000	10,000	250,000

**Total**	**100,000**		**3,250,000**	**100,000**	**3,500,000**
**APYL**			**32.5**		**35.0**

The high risk values for SBP and total cholesterol were 140 mmHg and 5.5 mmol/l respectively [19]. The target values for SBP and total cholesterol were the target levels published by the Australian National Heart Foundation (NHF)--120 mmHg and 4 mmol/l respectively [20,21]. For smoking the 'target' was taken as the absence of smoking, so the risk equations were recalculated with current smokers changed to non-smokers. The population prevalence of each risk factor above its target level was estimated by calculating the corresponding proportion from the AusDiab survey weighted using the survey weights.

The risk factor prevalence estimates and the baseline and scenario potential life years per person estimates were age-standardised using the Major City population as the standard population. This allowed direct comparisons between geographic regions.

Calculations of the proportions of risk and of people in each percentile group were based on relatively small numbers in some region, age, and sex groups leading to excessive variability in the resulting proportions. Thus, we applied a LOESS non-parametric smoothing procedure to the proportions [22]. We used bootstrapping to construct confidence intervals for all estimates presented in this paper with the exception of those arising from the Cox proportion hazards regression models, where standard errors and confidence intervals were generated by the regression modelling procedure [23]. The modelling, smoothing and regression calculations were conducted using SAS statistical software (SAS version 9.1.3. SAS Institute Inc.).

## Results

### Risk factor prevalence

Table 3 gives the estimated age-standardised proportion of people with high risk factor value in the Australian population by geographic region, and the standardised prevalence ratio (SPR) between Major Cities and each other region.

**Table 3:** Prevalence of high values of risk factors by geographic region, age standardised

		Major City		Inner Regional	Outer Regional/Remote
**Males**					
Smoker	Prevalence	14.2% (11.7%, 16.2%)		14.8% (11.1%, 17.9%)	19.6% (15.2%, 24.5%)
	SPR	1		1.04 (0.74, 1.35)	1.38 (1.06, 1.93)
Cholesterol	Prevalence	53.2% (49.8%, 55.7%)		54.7% (50.2%, 59.0%)	61.1% (56.1%, 65.5%)
	SPR	1		1.03 (0.92, 1.12)	1.15 (1.01, 1.27)
Systolic blood pressure	Prevalence	32.6% (29.9%, 35.9%)		30.6% (26.8%, 34.4%)	38.0% (32.7%, 43.5%)
	SPR	1		0.94 (0.84, 1.18)	1.17 (0.91, 1.28)

**Females**					
Smoker	Prevalence	9.3% (7.6%, 11.2%)		11.4% (8.5%, 14.0%)	13.0% (9.5%, 15.9%)
	SPR	1		1.22 (0.89, 1.63)	1.39 (1.00, 1.90)
Cholesterol	Prevalence	56.3% (53.1%, 59.6%)		55.8% (51.9%, 59.6%)	60.6% (55.7%, 64.7%)
	SPR	1		0.99 (0.91, 1.09)	1.08 (0.97, 1.18)
Systolic blood pressure	Prevalence	27.4% (24.7%, 30.1%)		27.2% (23.8%, 30.8%)	26.1% (22.1%, 30.4%)
	SPR	1		0.99 (0.75, 1.22)	0.95 (0.76, 1.26)

Notes

1. Proportion of the Australian population at ages 40 and over with high values of risk factors by geographic region, age standardised.

2. Prevalence estimated from AusDiab survey with 95% confidence intervals in brackets.

3. Age standardised prevalence rates and Standardised Prevalence Ratios (SPR) calculated using Major City population as the standard population.

4. The high risk values for systolic blood pressure and total cholesterol were 140 mmHg and 5.5 m mol/l respectively

The highest proportion was for cholesterol, with more than 50% of the population having a high cholesterol value for both sexes in each region--ranging from 53.2% (95%CI: 50.2%- 56.4%) in men in Major Cities to 61.1% (95%CI: 55.7%-66.1%) also in men but in the combined Outer Regional/Remote region.

For men, the prevalence of smoking and high cholesterol rose between Major Cities and Inner Regional areas and rose even further in Outer Regional/Remote areas, though the confidence intervals suggest that the differences were only statistically significant in the Outer Regional/Remote areas. The SPR values for cholesterol between Major Cities and Inner Regional and Outer Regional/Remote Areas were 1.03 (95%CI: 0.92-1.12) and 1.15 (95%CI: 1.01-1.27) respectively. The corresponding SPR values for smoking were 1.04 (95%CI: 0.80-1.37) and 1.38 (95%CI: 1.06-1.83). The prevalence of high SBP fell between Major Cities and Inner Regional areas but rose again in Outer Regional/Remote areas. However the SPR values all had confidence intervals containing 1--suggesting that none of the differences was statistically significant.

For women, the prevalence of smoking showed a similar pattern to that for men with SPR values of 1.22 (95%CI: 0.92-1.67) and 1.39 (95%CI: 1.00-1.90). The prevalence of high cholesterol fell between Major Cities and Inner Regional areas but rose again in Outer Regional/Remote areas. The prevalence of high SBP fell between Major Cities and Inner Regional areas and remained low in Outer Regional/Remote areas. The SPR values for both cholesterol and SBP had confidence intervals containing 1--suggesting that none of the differences was statistically significant.

### The SCORE equation as a predictor of all causes mortality

There were 246 deaths prior to 1 June 2004 among the 8,534 survey participants included in our modelling--154 men and 92 women. Of those participants aged 65 and over, there were 191 deaths--116 men and 75 women.

The results of fitting the Cox proportional hazards regression model to predict all-cause mortality using the SCORE index as a predictor variable are given in Tables 4, 5, 6 and 7. The SCORE index is a strongly significant predictor of mortality for ages 40 and over for both men and women (p = 0.012 for men and p = 0.0037 for women). The adjusted Hosmer-Lemeshow goodness of fit statistic shows no evidence of lack of fit in either equation (*χ*^2 ^value 6.58, p = 0.68 for men and *χ*^2 ^value 9.37, p = 0.40 for women) and the C statistics show good predictive power with values of 83% for men and 84% for women--C values above 80% are generally regarded as demonstrating excellent predictive power [24].

**Table 4:** Results of Cox proportional hazards regression model, males aged 40 and over

Parameter	DF	Estimate	Standard Error	Chi-Square	Pr > ChiSq	Hazard Ratio	95% confidence limits	
Score	1	1.58	0.63	6.29	0.0121	4.83	1.41	16.55
Age 40-44	1	-4.31	1.03	17.58	< .0001	0.01	0.00	0.10
Age 45-49	1	-2.35	0.42	31.09	< .0001	0.10	0.04	0.22
Age 50-54	1	-3.10	0.55	31.99	< .0001	0.05	0.02	0.13
Age 55-59	1	-1.99	0.39	25.54	< .0001	0.14	0.06	0.30
Age 60-64	1	-1.29	0.32	16.57	< .0001	0.28	0.15	0.51
Age 65-69	1	-1.02	0.28	13.18	0.0003	0.36	0.21	0.63
Age 70-74	1	-0.53	0.24	5.11	0.0238	0.59	0.37	0.93

**Adjusted Hosmer-Lemeshow goodness of fit**								

Chi-Square		DF		Pr > ChiSq				
6.58		8		0.68				

Notes

1. Results of a Cox proportional hazards regression model predicting risk of death prior to 1 June 2004 among male AusDiab participants aged 40 and over

2. Age group 75 and over taken as baseline group for the age parameter

C statistic: 83%

**Table 5:** Results of Cox proportional hazards regression model, females aged 40 and over

Parameter	DF	Estimate	Standard Error	Chi-Square	Pr > ChiSq	Hazard Ratio	95% confidence limits	
score	1	2.32	0.80	8.42	0.0037	10.20	2.13	48.95
Age 40-44	1	-3.23	0.75	18.41	<.0001	0.04	0.01	0.17
Age 45-49	1	-2.80	0.63	19.72	<.0001	0.06	0.02	0.21
Age 50-54	1	-2.22	0.51	18.81	<.0001	0.11	0.04	0.30
Age 55-59	1	-2.55	0.63	16.57	<.0001	0.08	0.02	0.27
Age 60-64	1	-2.09	0.55	14.27	0.0002	0.12	0.04	0.37
Age 65-69	1	-1.35	0.42	10.50	0.0012	0.26	0.12	0.59
Age 70-74	1	-0.27	0.28	0.90	0.3433	0.76	0.44	1.33

**Adjusted Hosmer-Lemeshow goodness of fit**								

Chi-Square		DF		Pr > ChiSq				
9.37		8		0.40				

Notes

1. Results of a Cox proportional hazards regression model predicting risk of death prior to 1 June 2004 among female AusDiab participants aged 40 and over

2. Age group 75 and over taken as baseline group for the age parameter

C statistic: 84%

**Table 6:** Results of Cox proportional hazards regression model, males aged 65 and over

Parameter	DF	Estimate	Standard Error	Chi-Square	Pr > ChiSq	Hazard Ratio	95% confidence limits	
score	1	1.51	0.64	5.59	0.0181	4.54	1.30	15.94
Age 65-69	1	-1.03	0.28	13.51	0.0002	0.36	0.21	0.62
Age 70-74	1	-0.54	0.24	5.24	0.022	0.58	0.37	0.93

**Adjusted Hosmer-Lemeshow goodness of fit**								

Chi-Square		DF		Pr > ChiSq				
12.00		8		0.21				

Notes

1. Results of a Cox proportional hazards regression model predicting risk of death prior to 1 June 2004 among male AusDiab participants aged 65 and over

2. Age group 75 and over taken as baseline group for the age parameter

C statistic: 67%

**Table 7:** Results of Cox proportional hazards regression model, females aged 65 and over

Parameter	DF	Estimate	Standard Error	Chi-Square	Pr > ChiSq	Hazard Ratio	95% confidence limits	
score	1	2.34	0.80	8.49	0.0036	10.33	2.15	49.67
Age 65-69	1	-1.35	0.42	10.48	0.0012	0.26	0.12	0.59
Age 70-74	1	-0.27	0.28	0.89	0.3465	0.77	0.44	1.34

**Adjusted Hosmer-Lemeshow goodness of fit**								

Chi-Square		DF		Pr > ChiSq				
16.11		8		0.06				

Notes

1. Results of a Cox proportional hazards regression model predicting risk of death prior to 1 June 2004 among female AusDiab participants aged 65 and over

2. Age group 75 and over taken as baseline group for the age parameter

C statistic: 71%

The SCORE index is still a strong predictor of mortality when the regression is restricted to ages 65 and over (p = 0.018 for men and p = 0.0036 for women). The adjusted Hosmer-Lemeshow goodness of fit statistic shows no evidence of lack of fit in either equation (*χ*^2 ^value 12.00, p = 0.21 for men and *χ*^2 ^value 16.11, p = 0.06 for women) and the C statistics show reasonable predictive power with values of 67% for men and 71% for women.

A similar analysis with follow-up of death to 2008 was also done to test the sensitivity of the conclusions to the choice of follow-up date (results not shown). The SCORE statistic also performed well as a predictor of all-cause mortality with this follow-up date.

### Modelled regional life expectancy

The survival curves and associated life expectancies were calculated separately for each age-sex-region-percentile group. However, for ease of presentation, we have illustrated the results of this modelling by presenting them at age 40 for the highest and lowest deciles of projected mortality risk.

**Figure 1:**
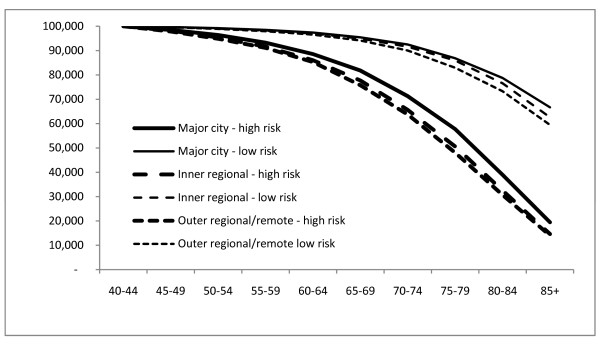
**Survival by region, males, Australia, 2001-2006**. Number per 100,000 surviving at each age above 40 by geographic region for the lowest and highest deciles of mortality risk, Males, Australia, 2001-2006.

**Figure 2:**
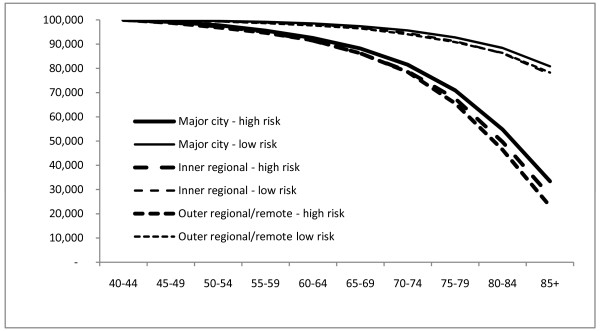
**Survival by region, females, Australia, 2001-2006**. Number per 100,000 surviving at each age above 40 by geographic region for the lowest and highest deciles of mortality risk, Females, Australia, 2001-2006.

Figures 1 and 2 present the number per 100,000 from the life table surviving at each age over 40 for the lowest and highest deciles of predicted mortality risk by region. Survival is better for women than men in each region at each risk level. Survival is also better in Major Cities than Outer Regional/Remote areas for men and women at each risk level.

**Figure 3:**
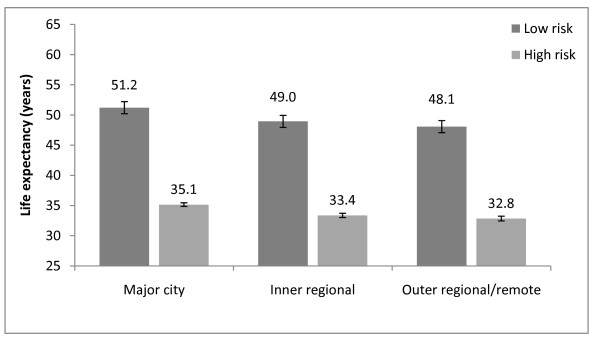
**Life expectancy by geographic region, Males, Australia, 2001-2006**. Life expectancy at age 40 by geographic region for the lowest and highest deciles of mortality risk, Males, Australia, 2001-2006.

**Figure 4:**
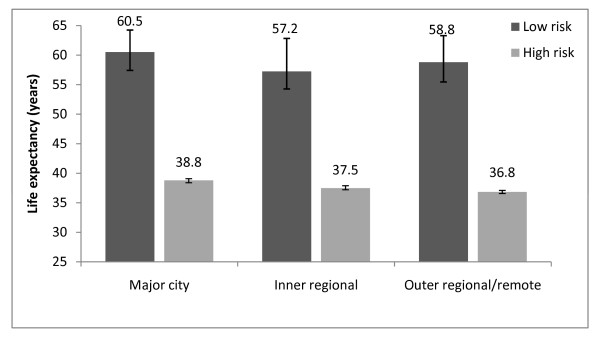
**Life expectancy by geographic region, Females, Australia, 2001-2006**. Life expectancy at age 40 by geographic region for the lowest and highest deciles of mortality risk, Females, Australia, 2001-2006.

Figures 3 and 4 present life expectancies at age 40 years for the lowest and highest deciles of mortality risk in each region. For men in both low and high risk groups, there is a relatively steady fall in life expectancy across the Major City, Inner Regional and Outer Regional/Remote areas. The life expectancy difference between Major Cities and Outer Regional/Remote areas was 3.1 years for the low-risk group (95%CI: -0.9, 5.9) and 2.3 years for the high-risk group (95%CI: 1.8, 2.8) (data not shown).

For women at low risk, there is no consistent pattern in life expectancy between the Major City, Inner and Outer Regional/Remote areas--with life expectancy falling between Major Cities and Inner Regional areas but rising again between Inner and Outer Regional areas. Women at high risk are more similar to their male counterparts in having a relatively steady fall in life expectancy across the Major City, Inner and Outer Regional/Remote areas. The difference in female life expectancy for those in the high risk group between Major Cities and Outer Regional/Remote areas is 2.0 years (95%CI: 1.5, 2.3) (data not shown).

### The effect of modifiable risk factors on average potential years of life (APYL) in each region

Table 8 presents the APYL per person (age standardised) for the Australian population in 2006 by sex and region for the baseline and each risk factor target scenario and for all three risk factor scenarios together, and the differences in APYL between the baseline and each scenario.

**Table 8:** APYL per person at age 40 by geographic region for selected risk factor scenarios

	Major city	Inner regional	Outer regional/remote
**Males**	**Years (95% CI)**	**Years (95% CI)**	**Years (95% CI)**
Baseline	29.4 (29.1, 29.8)	27.9 (27.6, 28.3)	27.0 (26.7, 27.8)
Total cholesterol < 5.5	31.9 (31.4, 32.5)	30.5 (29.7, 31.3)	30.0 (29.2, 31.3)
SBP < 140	31.6 (31.0, 32.1)	29.8 (29.2, 30.7)	29.4 (28.9, 30.6)
No smoking	30.4 (30.1, 30.8)	29.0 (28.6, 29.4)	28.6 (28.2, 29.4)
1-3 combined	35.7 (34.7, 36.6)	33.9 (32.9, 35.4)	34.4 (32.8, 36.5)

**Females**	**Years (95% CI)**	**Years (95% CI)**	**Years (95% CI)**
Baseline	32.9 (32.3, 33.5)	31.5 (30.7, 32.2)	31.1 (30.4, 32.0)
Total cholesterol < 5.5	35.4 (34.5, 36.5)	33.9 (32.7, 35.6)	34.1 (32.9, 35.3)
SBP < 140	35.4 (34.5, 36.5)	33.8 (32.4, 34.9)	33.6 (32.3, 35.3)
No smoking	33.8 (33.3, 34.7)	32.6 (32.1, 33.3)	32.6 (31.9, 33.4)
1-3 combined	39.3 (38.0, 40.7)	37.5 (36.2, 39.4)	38.2 (36.2, 40.4)

**Difference from baseline**			
**Males**	**Years (95% CI)**	**Years (95% CI)**	**Years (95% CI)**
Total cholesterol < 5.5	2.5 (1.8, 3.2)	2.6 (1.7, 3.5)	3.0 (1.7, 4.2)
SBP < 140	2.2 (1.5, 2.9)	1.9 (1.0, 2.7)	2.4 (1.3, 3.4)
No smoking	1.0 (0.5, 1.5)	1.1 (0.6, 1.7)	1.5 (0.7, 2.3)
1-3 combined	6.3 (5.2, 7.3)	6.0 (4.6, 7.3)	7.3 (5.4, 9.3)

**Females**	**Years (95% CI)**	**Years (95% CI)**	**Years (95% CI)**
Total cholesterol < 5.5	2.6 (1.4, 3.7)	2.4 (0.8, 4.1)	3.0 (1.5, 4.5)
SBP < 140	2.5 (1.3, 3.7)	2.3 (0.9, 3.7)	2.5 (0.8, 4.2)
No smoking	1.0 (0.0, 1.9)	1.1 (0.2, 2.1)	1.5 (0.4, 2.6)
1-3 combined	6.4 (5.0, 7.9)	6.1 (4.4, 7.8)	7.1 (4.8, 9.3)

Note: Age standardised APYL calculated using Major City population as the standard population

Setting high values of each risk factor to its target increases the APYL in each region. The risk factor associated with the largest rise in APYL across all regions was high cholesterol for both men and women--with an increase in APYL of between 2.5 and 3.0 years for men and between 2.4 and 3.0 years for women. Setting all three risk factors together to no higher than their target levels led to an increase in APYL of between 6.0 and 7.3 years for men and 6.1 and 7.1 years for women. The increase was greatest in Outer Regional/Remote areas for each risk factor except for SBP in women, where the Major City and Outer Regional/Remote increases were equal.

The effect of setting each risk factor to its target on the difference in APYL between Major Cities and each other region largely followed the prevalence distribution (Table 9). For men the prevalence of smoking and high cholesterol rose by a small amount between Major Cities and Inner Regional areas and rose again by a larger amount in Outer Regional areas. The prevalence of high SBP fell between Major Cities and Inner Regional areas but rose again in Outer Regional areas. Accordingly eliminating smoking and high cholesterol was associated with a decrease in the APYL differential between Major Cities and Inner Regional areas (by 6.3% and 8.8% respectively). Eliminating high values of each of the three risk factors separately (smoking, cholesterol and SBP) was associated with a decrease in the APYL differences between Major Cities and Outer Regional/Remote areas by 21.4%, 20.3% and 7.7% respectively. Overall the elimination of high values of cholesterol and SBP and the elimination of smoking together was associated with a fall in the differential between Major Cities and Outer Regional/Remote areas (by 45.4%) but not with a fall in the differential between Major Cities and Inner Regional areas.

**Table 9:** Difference in APYL per person at age 40 between major cities and other regions

	Inner regional	Outer regional/remote
**Males**	**Years (95% CI)**	**Years (95% CI)**
Baseline	1.5 (0.9, 1.9)	2.4 (1.7, 2.9)
Total cholesterol < 5.5	1.4 (0.6, 2.4)	1.9 (0.4, 3.2)
SBP < 140	1.8 (1.0, 2.6)	2.2 (1.1, 3.0)
No smoking	1.4 (0.9, 2.0)	1.9 (1.0, 2.3)
1-3 combined	1.8 (-0.1, 3.4)	1.3 (-1.2, 3.1)

**Females**	**Years (95% CI)**	**Years (95% CI)**
Baseline	1.4 (0.5, 2.7)	1.7 (0.9, 2.6)
Total cholesterol < 5.5	1.5 (-0.3, 2.9)	1.3 (-0.3, 3.1)
SBP < 140	1.6 (0.4, 3.4)	1.7 (-0.3, 3.5)
No smoking	1.2 (0.3, 2.2)	1.2 (0.2, 2.2)
1-3 combined	1.8 (-0.8, 3.5)	1.1 (-1.6, 4.0)

**% difference associated with each risk factor**		
**Males**		
Total cholesterol < 5.5	8.8%	20.3%
SBP < 140	-21.0%	7.7%
No smoking	6.3%	21.4%
1-3 combined	-19.6%	45.4%

**Females**		
Total cholesterol < 5.5	-9.8%	24.0%
SBP < 140	-11.9%	-0.1%
No smoking	12.3%	29.4%
1-3 combined	-26.7%	35.6%

Notes

1. Age standardised APYL calculated using Major City population as the standard population

2. Negative proportions indicate cells where APYL difference increased for the risk factor scenario

For women, with the exception of smoking, the prevalence of high risk factor values fell between Major Cities and Inner Regional areas. The prevalence of smoking and high cholesterol rose between Major Cities and Outer Regional/Remote areas but the prevalence of high SBP in Outer Regional/Remote areas remained below that of Major Cities. Accordingly smoking was the only risk factor whose elimination was associated with a decrease in the APYL differential between Major Cities and Inner Regional areas (by 12.3%) while eliminating each of smoking and high cholesterol was associated with a decrease in the differential between Major Cities and Outer Regional/Remote areas (by 29.4% and 20.4% respectively). Overall the elimination of high values of cholesterol and SBP and the elimination of smoking together was associated with a fall in the differential between Major Cities and Outer Regional/Remote areas (by 35.6%) but not with a fall in the differential between Major Cities and Inner Regional areas.

## Discussion

Eliminating smoking and setting high values of total cholesterol and SBP to their recommended target levels increased average potential years of life per person by between 6.0 and 7.3 years for men and 6.1 and 7.1 years for women. The increase was greatest in Outer Regional/Remote areas for all risk factors except for SBP in women, where the Major City increase was equal to the Outer Regional/Remote area increase.

Mortality differences between regions affect men at all risk levels but mainly apply to higher risk women. Eliminating smoking would reduce mortality differences between Major Cities and each other region for both men and women. In addition, reducing high cholesterol would reduce mortality differences between Major Cities and each other region for men. Reducing high cholesterol in women or high SBP in men would reduce the mortality difference between Major Cities and Outer Regional/Remote areas but not Inner Regional areas. The combined effect of reducing high cholesterol and high SBP and eliminating smoking would reduce the APYL difference between Major Cities and Outer Regional/Remote areas by 45.4% for men and 35.6% for women.

Previous research has found a complex relationship between geographic variation in risk factors and mortality. Papastergiou and colleagues found that low economic activity and access to health services were significant drivers of regional variation in all-cause mortality in Greece [4]. Romeri and colleagues studied regional variation in mortality in England and Wales and found that while mortality increased with deprivation, the relationship was strongest for smoking-related causes--suggesting an unmeasured role for smoking [5]. Men et al. analysed regional trends in Russian mortality and concluded that fluctuations in mortality correlated strongly with underlying economic and societal factors but that risk factors (particularly alcohol) played a part at an individual level [6]. Bassuk and colleagues studied mortality in the elderly in four US communities and concluded that while individual characteristics such as health risk factors played a role, it was also important to consider community attributes that mediate or modify the pathways through which socioeconomic conditions may influence health [7]. Our study provides quantification in the Australian population of the effect of individual risk factors. However, as noted by the researchers quoted here, the individual risk factors must be viewed in the context of broader societal and economic influences. Hence, while our study did not address health services, societal and economic factors, these broader factors may be the principal drivers of regional mortality differentials while the health risk factors may act as mediators.

The WHO Comparative Risk Assessment project developed methods for comparing the disease burden attributable to different health risk factors in a standardised way. These methods are based on the use of population attributable fractions (PAF) with a consistent theoretical framework that uses the 'hypothetical minimum' as the counterfactual against which burden due to a risk is calculated. They also include continuous risk variables accounting for the full range of risk from elevated blood pressure and serum cholesterol, rather than defining thresholds for hypertension and hypercholesterolaemia [25]. These methods have been used to develop estimates of the Australian disease burden for the major health risk factors [17]. In principle, these methods could be used to model the difference in burden between regions by examining differences in risk factor prevalence and inferring differences in disease burden. However, our methods have the strength of directly modelling the absolute effects of specific risk factors on the inter-regional difference in all-cause mortality. PAF's are typically derived for specific causes of death and so would require separate modelling for each cause related to each risk factor and then aggregating across different causes. Our methods do, however, have the limitation of using risk factor threshold values rather than using the whole risk factor distribution. This may lead to an underestimate of the health impact of the risk factor and hence a conservative estimate of its impact on inter-regional mortality differences.

The major strength of our modelling approach is the ability to apportion mortality to percentiles of mortality risk based on measured rather than self-reported cholesterol and blood pressure values and total population and mortality counts. This allows us to model the effect of changing risk factor profiles on the distribution of risk within the population.

One limitation of our study is the use of the SCORE equation as a proxy for all-causes mortality risk. Our study requires an all-causes mortality risk prediction equation based on the modifiable risk factors measured in our population based sample. Our literature search found two such equations in the literature which could potentially be suitable for application to our population survey data--one based on the Multiple Risk Factor Intervention Trial (MRFIT) study developed by Kannel et al. [26] and one based on the Aerobics Center Longitudinal Study at the Cooper Clinic in Dallas, Texas (the Cooper Clinic Mortality Risk Index) developed by Janssen et al. [27]. However, both of these equations were developed for men only and we need to be able to apply our risk equation to both men and women. Further, the MRFIT equation used diastolic blood pressure as its blood pressure measure rather than the more commonly used systolic blood pressure and the Cooper Clinic index incorporated an exercise stress test of cardiorespiratory fitness which was not available in our population based sample. So neither equation was suitable for our study.

Cardiovascular disease is the leading cause of death in Australia--comprising 34% of all deaths registered in 2008 [28]. Further, tobacco smoking as well as being a leading risk factor for cardiovascular disease (CVD) is also a leading risk factor for death from a wide range of other causes [7]. This suggests that a cardiovascular mortality risk prediction equation incorporating tobacco use among its predictor variables such as the SCORE index may be a suitable proxy for an all-causes mortality risk prediction equation. Aktas et al. examined the SCORE equation as a predictor of all cause mortality risk in a sample of 3,554 asymptomatic adults (2871 men and 683 women) aged 50 - 75 years at the Cleveland Clinic Foundation in Cleveland, Ohio [3]. They found that the SCORE index was strongly predictive of all-cause mortality in their sample. Further, they found that SCORE was a considerably better predictor of all-cause mortality than the Framingham risk score, which is the other most commonly used CVD risk score. We used a similar approach to Aktas et al. in using a Cox proportional hazards regression model to predict all-cause mortality within our population based sample with the SCORE as the predictor variable. Our Cox regression analysis showed that the SCORE index was a good predictor of all-causes mortality for both men and women at ages 40 and over and at ages 65 and over. The original SCORE index was derived for men and women aged 45 - 64. Our regression for people aged 40 and over supports our use of the SCORE index as a proxy for all-cause mortality risk in our risk percentiles model. Our regression for people aged 65 and over demonstrates that the SCORE index can be used as a proxy for all-causes mortality risk at older ages than the original group for which it was derived.

Another limitation is that the AusDiab survey participants are known to have lower mortality risk than the general Australian population, despite being drawn from a population-based random sample. However, our study relies on the ordering of risk within the population rather than the absolute level of risk and so should be relatively robust to this limitation.

A further limitation is that our study excludes Very Remote areas and does not provide separate results for Remote areas. This is a limitation of a population based modelling approach as less than three per cent of the Australian population live in Remote and Very Remote areas. Hence the numbers of people and of deaths are too small in these areas to support separate estimates using our modelling approach.

The study is limited to those risk factors incorporated in the SCORE risk prediction equation--smoking, blood pressure and cholesterol. Tobacco and blood pressure are the two leading risk factors associated with disease burden in Australia and cholesterol is the fifth (after obesity and physical inactivity), so these risk factors are appropriate for this study [17]. The exclusion of other risk factors renders our study results conservative in estimating the total contribution of modifiable risk factors to life expectancy differentials. Further work in this area would benefit from developing an all-causes mortality risk prediction equation incorporating further risk factors.

Our study is an 'ecological' study in that it only examines risk factor prevalence at the regional level and hence neglects the distribution of risk factors and life expectancy within these regions. This may also lead to underestimation of the contribution of the risk factors to the mortality differential.

Our modelling is aimed at quantifying the contribution of modifiable health risk factors to regional mortality differentials. Hence our estimates represent an upper bound to the gains that could be made from interventions targeting these risk factors rather than a projection of the actual gains from any specific health promotion activity. However, they do demonstrate that there are potentially substantial gains in health equity which could arise from addressing modifiable health risk factors in outer regional and remote areas.

## Conclusions

Australian mortality rates are higher in regional and remote areas than in major cities and the associated health inequities have been identified as significant human rights issues for Australia [8]. Our results suggest that health intervention programs aimed at smoking, blood pressure and total cholesterol could have a substantial impact on mortality inequities at least for Outer Regional/Remote areas.

## Competing interests

The authors declare that they have no competing interests.

## Authors' contributions

CES conceived of the study, carried out the statistical analyses and drafted the manuscript. HM programmed the SAS macros to calculate the Harrel's C and modified Hosmer-Lemeshow statistics. CES, HM, AP, HW and JJM collaborated on the study design. DJM and JES provided the population survey data and advice on its interpretation. All authors participated in revisions of the draft manuscript. All authors read and approved the final manuscript.

## Pre-publication history

The pre-publication history for this paper can be accessed here:

http://www.biomedcentral.com/1471-2458/12/79/prepub

## Supplementary Material

Additional file 1 **Appendix**: the risk percentiles model. Click here for file
