# Supplementary material for: The effect of modifiable risk factors on geographic mortality differentials: a modelling study
Source: BMC Public Health. 2012 Jan 25;12:79. doi: 10.1186/1471-2458-12-79 (PMC3349565; doi:10.1186/1471-2458-12-79)
Supplement: Additional file 1 — Appendix: the risk percentiles model. [file 1471-2458-12-79-S1.DOCX]

# Appendix: the risk percentiles model

## Introduction

The risk percentiles model is an adaptation of an earlier coronary heart disease (CHD) prevention model.([1](#_ENREF_1)) It is a mathematical model capable of projecting the lifetime risk of death from any cause among individuals in different percentiles of integrated mortality risk. The primary modelling strategy is the distribution of absolute short-term mortality risk within the Australian population projected using risk prediction equations from published studies. The modelling assumption behind this is that deaths will follow the same distribution as the projected risk and so we can use the distribution of projected risk to allocate observed deaths to percentiles of risk. The basis of the model is the construction of separate life tables for people at different levels of mortality risk and the generation of risk-specific survival curves from each of these life tables.

## Data sources

This application of the risk percentiles model requires:

1. a nationally representative population survey with information collected on health risk factors as well as age, sex and geographic region;
2. a published risk prediction equation linking health risk factors to the risk of death from any cause; and
3. national population counts and counts of deaths from any cause classified by age, sex and geographic region.

In this application of the model, the national population survey was the baseline survey of the Australian Diabetes, Obesity and Lifestyle (AusDiab) study.([2](#_ENREF_2)) This was a cross-sectional, national, population-based survey of 11,247 adults aged ≥25 years in 1999–2000, comprising a household interview and biomedical examination at a testing site. Among the information collected was seated systolic and diastolic blood pressure; self-reported cigarette smoking status; and serum total cholesterol. Of the 11,247 survey participants 8,706 were aged 40 or over. Of these 8,534 had complete data for systolic and diastolic blood pressure; self-reported cigarette smoking status; and serum total cholesterol—3,880 males and 4,654 females.

The risk prediction equation was the index developed by the SCORE (Systematic COronary Risk Evaluation) project.([3](#_ENREF_3)) National population and death counts classified by age, sex and geographic region for 2001 to 2006 were supplied by the Australian Institute of Health and Welfare (AIHW). The population counts were derived from the Australian Bureau of Statistics (ABS) mid-year population estimates. Deaths data were derived from the AIHW National Mortality Database comprising all deaths registered in Australia.

## Phases of the model

The model proceeded in two phases. The first phase involved allocating observed deaths to percentiles of mortality risk. The model applied the risk prediction equation to the population survey to project the levels of risk that divide the population into percentiles of mortality risk. By definition, the overall population will be evenly spread between the percentiles of risk, but the absolute risk will be higher in higher percentiles. The observed counts of deaths were then allocated to each percentile of risk in proportion to the modelled level of absolute risk in each percentile, enabling mortality rates to be calculated for each percentile of risk. These mortality risks were then used to calculate life tables for each percentile of risk.

The second phase involved modelling the effect of risk factors on regional mortality. AusDiab participants with high values of each risk factor had their values set to target values, thus creating a synthetic sample of people with risk factors at or below their target levels. These participants were then re-allocated to lower risk percentiles based on their new risk factor values. This, when weighted using the AusDiab survey weights, provided a modelled estimate of the shift in the total Australian population between each percentile group when high risk factor values were lowered to their target values. This second phase was applied to the 2006 Australian population.

The application of each of these model phases is described in detail below.

## Average Potential Years of Life (APYL) per person

The overall outcome measure used in this model was *Average Potential Years of Life (APYL)* per person. The life expectancy in each risk percentile group was calculated for each age-sex-region group using the life tables derived in phase 1 of the model. This was multiplied by the corresponding 2006 population count to get the projected total life years in each risk percentile for each age-sex-region group. This was summed over the percentiles and ages within each region and divided by the regional population count to calculate a *baseline APYL per person* for each sex in each region. Similar APYL values were calculated for the synthetic populations derived by setting each of the risk factors to at or below their target values as described above. These were designated the scenario APYL per person.

## Phase 1: Allocating deaths to percentiles of risk

Phase 1 of the risk percentiles model was used to derive an average life expectancy at each percentile of projected mortality risk based on the SCORE equation. The Australian disease burden associated with the modifiable risk factors incorporated in our risk prediction equation is very small below age 40, so our modelling was applied to ages 40 and over.([4](#_ENREF_4)) The steps involved in the calculation, within each age, sex and regional group, were as follows:

1. *Divide the Australian population into mortality risk percentiles.*

The SCORE equation was applied to the AusDiab data to calculate the five-year probability of death for each individual in the survey (their *risk score*). The values of the risk score which divide the population into percentiles were then calculated, weighted with the survey weights so that these could be taken as estimates for the total Australian population. Each survey participant was allocated to a risk percentile using their risk score and a total risk score calculated for each percentile by summing the individual risk scores for the survey participants in that percentile.

1. *Use the relativities of the risk scores to allocate observed deaths to risk percentiles*

The national count of deaths summed across 2001 to 2006 was allocated to the risk percentiles using the ratios of the aggregate risk score between percentiles as follows. The relative mortality risks were calculated as the ratio of the total risk scores between percentiles and the observed deaths were allocated to the percentiles according to these ratios. For example, if one percentile group had a risk score twice that of another group then the deaths were allocated between them in the ratio 2 to 1. When divided by the population count (assumed evenly spread between risk percentiles) this gives us the mortality rate for each risk percentile group within each age, sex, regional group.

Calculations of the proportion of deaths in each percentile group were based on relatively small numbers in some region, age, sex groups and so the resulting proportions were subject to some variability. To overcome this, we applied a LOESS non-parametric smoothing procedure(5) to the estimated proportions using the LOESS procedure in the SAS statistical software package.(6)

1. *Use these mortality rates to construct sex-specific life tables and survival curves for each risk percentile within each regional group.*

After step 2, we had a set of age specific mortality rates by sex for each percentile group within each region. We applied standard life table techniques(7) using these mortality rates to construct a sex-specific life table for each percentile group within each region. We used these life tables to generate sex-specific survival curves and life expectancies for each percentile group within each region.

1. *Use bootstrapping to construct confidence intervals for life table quantities*

There are closed-form equations for estimating confidence intervals for quantities derived from life tables, but they are not appropriate here as the major uncertainty in any modelled value arises from the uncertainties inherent in the modelling process. Instead a measure of the variability in the modelled estimates was derived by applying bootstrapping techniques to the AusDiab sample which is the basis of the modelled estimates.

The above calculations were done at the percentile risk level but were grouped into deciles of mortality risk for ease of reporting.

## Phase 2: Modelling the effect of risk factors on inter-regional mortality differentials

As noted above, the effect of risk factors on inter-regional mortality differentials was modelled for the 2006 Australian population. The steps involved in the calculation were as follows:

1. *Calculate a baseline average potential years of life (APYL) per person for each region.*

The average life expectancy by sex for each percentile group within each region was extracted from the life tables calculated in phase 1. This life expectancy was multiplied by the corresponding 2006 population count to get the projected total life years by sex for each percentile group within each region. This was summed over the percentiles and ages within each region and divided by the regional population count to calculate a *baseline APYL per person* by sex for each region.

1. *Set high values of each risk factor separately to a ‘target’ value.*

The risk factors modelled in this paper were those in the SCORE equation which are potentially modifiable—smoking, total cholesterol and systolic blood pressure (SBP).([3](#_ENREF_3)) We returned to the AusDiab survey data to model the effect of setting high values of each risk factor separately to a ‘target’ value. For example, in examining blood pressure each survey participant with a SBP greater than or equal to 140 mmHg had his/her risk score recalculated assuming a SBP of 120—creating a synthetic sample of people with SBP below high risk levels.

1. *Re-allocate participants to risk percentiles.*

Those participants who had their risk scores reduced in the synthetic sample were then re-allocated to a lower risk percentile group on the basis of their revised risk score but using the existing risk score cutoffs as calculated in step 1 of phase 1. Note that this implies that the ‘percentile’ groups in the synthetic sample are no longer true percentiles. The participants who had their risk scores reduced moved to a lower ‘percentile’ group. Hence the participants in the synthetic sample were no longer evenly distributed between the groups. So in the description of phase 2 of the modelling we will refer to these groups as the *risk groups*.

This re-allocation, when weighted using the AusDiab survey weights, provided a modelled estimate of the shift in the total Australian population between each risk group which would arise if all the high values of the risk factor in the population were reduced to the target value.

For example, in examining blood pressure, our synthetic sample contained participants whose true SBP value was 140 mmHg or greater and whose synthetic SBP value was 120 mmHg. Each of these participants was re-allocated to a lower risk group by re-calculating their risk score using the synthetic SBP value of 120 and allocating them to a new risk group using this re-calculated risk score.

1. *Calculating the scenario APYL per person for each risk factor.*

We have a projected average life expectancy by sex and age for each risk group in each region calculated from step 4 of phase 1. We applied the modelled population shift from step 3 above to the 2006 Australian population counts to derive a modelled estimate of the population by age and sex for each risk group in each region. We multiplied the average life expectancy for each percentile-age-sex-region group by the new population count projected to be in that group under each scenario. This was summed over the percentiles and ages within each region and divided by the regional population count to calculate a *scenario APYL per person* for each region.

For example, we calculated the blood pressure scenario APYL by projecting the population shift between risk groups associated with lowering all raised blood pressure values to the target value. We then multiplied the average life expectancy in each risk group for each age-sex-region by the new population count projected to be in that group after all high SBP values had been set to the target value.

1. *Projecting the impact of each risk factor.*

The impact of each risk factor was measured by comparing the baseline APYL per person with the scenario APYL per person for that risk factor. For example, the impact of blood pressure was measured by comparing the baseline APYL per person with the blood pressure scenario APYL per person described in step 5 above.

1. *Use bootstrapping to construct confidence intervals*

As with phase 1, we applied bootstrapping techniques to construct confidence intervals for the APYL estimates.

## Illustrative example of the application of the model

This procedure is illustrated in table A1 with a hypothetical population of 100,000 people divided into risk quartiles (not percentiles) for ease of presentation. Column 2 presents the number of people in each quartile at baseline and column 3 is the average life expectancy from our model for each quartile. Column 4 presents the baseline lifeyears estimate, which is calculated by multiplying column 2 by column 3. If we sum column 4 and divide this by the total population, we get the baseline APYL per person (32.5 years). Column 5 presents the number of people allocated to each risk group after the survey participants have had their risk adjusted as described above. Column 6 represents the scenario lifeyears estimate and is calculated by multiplying column 3 by column 5. If we sum column 6 and divide this by the total population, we get the scenario APYL per person (35.0 years).

**Table A1: Modelling the impact of a risk factor illustrated with a hypothetical population**

| **Quartile of risk** | **Baseline population distribution** | **Average life expectancy** | **Baseline PYL** | **Scenario population distribution** | **Scenario PYL** |
| --- | --- | --- | --- | --- | --- |
| 1 | 25,000 | 40 | 1,000,000 | 40,000 | 1,600,000 |
| 2 | 25,000 | 35 | 875,000 | 30,000 | 1,050,000 |
| 3 | 25,000 | 30 | 750,000 | 20,000 | 600,000 |
| 4 | 25,000 | 25 | 625,000 | 10,000 | 250,000 |
| **Total** | **100,000** |  | **3,250,000** | **100,000** | **3,500,000** |
| **APYL** |  |  | **32.5** |  | **35.0** |

## References

1. McNeil JJ, Peeters A, Liew D, Lim S, Vos T. A model for predicting the future incidence of coronary heart disease within percentiles of coronary heart disease risk. Journal of Cardiovascular Risk 2001;8(1):31-7.

2. Dunstan DW, Zimmet PZ, Welborn TA, Cameron AJ, Shaw J, de Courten M, et al. The Australian Diabetes, Obesity and Lifestyle Study (AusDiab)--methods and response rates. Diabetes Research and Clinical Practice 2002;57(2):119-129.

3. Conroy RRM, Pyörälä KK, Fitzgerald AAP, Sans SS, Menotti AA, De Backer GG, et al. Estimation of ten-year risk of fatal cardiovascular disease in Europe: the SCORE project. European Heart Journal 2003;24(11):987-1003.

4. Begg S, Vos T, Barker B, Stevenson CE, Stanley L, Lopez AD. The burden of disease and injury in Australia 2003. cat. no. PHE 82. Canberra: AIHW; 2007.

5. Cleveland WS, Devlin SJ, Grosse E. Regression by local fitting. Journal of Econometrics 1988;37:87-114.

6. SAS Institute Inc. SAS 9.2 Language Reference: Concepts, Second Edition. Cary, NC: SAS Institute Inc.; 2010.

7. Chiang CL. The life table and its applications. Malabar, Florida: Robert E. Krieger Publishing Company, Inc.; 1984.

8. Australian Institute of Health and Welfare (AIHW). Heart, stroke and vascular diseases—Australian facts 2004. AIHW Cat. No. CVD 27. Canberra: AIHW and National Heart Foundation of Australia (Cardiovascular Disease Series No. 22); 2004.

9. National Heart Foundation of Australia (NHF). Guide to management of hypertension 2008. Updated August 2009. Web version. In: National Heart Foundation of Australia; 2009.

10. National Heart Foundation of Australia and The Cardiac Society of Australia and New Zealand. Lipid management guidelines - 2001. Medical Journal of Australia 2001;175:s57-s88.

11. Efron B, Tibshirani R. Bootstrap methods for standard errors, confidence intervals, and other measures of statistical accuracy. Statistical Science 1986;1(1):54-77.
